# On Malaria in Connection with Medical Topography

**Published:** 1848-08

**Authors:** R. S. Holmes

**Affiliations:** St. Louis, (late of the Medical Staff, U. S. A.)


*On malaria in connection with Medical Topography, by* R. S. Holmes, M. D., of St. Louis, (late of the Medical Staff, U. S. A.)—The subject is one to which but little attention has been paid, whilst the cause of miasmata, (nearly assimilated to it) has given rise to innumerable discussions; in the Northern States, Medical topography, though highly important, does not possess that absorbing interest that is attached to it in the South. There the health and happiness of a family, or a community, often depends on the circumstance of the position with regard to some adjacent swamp, or river bank, or low, moist alluvial ground, or a high ground with an impervious strata underneath, or a profuse hummack growth of timber, or the sedgy margin of some half stagnant stream; it becomes a matter of momentous interest to ascertain in what direction in the summer months the winds blow, and to know what is the nature of the soil to windward. The rude and uneducated settler in Georgia or Florida, builds his cabin on the highest knoll in the pine groves, though all beneath is the white sand of the south. If you ask him the reason, he tells you it is healthier there, though he probably goes half a mile to his little plantation; nature has been his teacher in the matter, and although he knows nothing of the theory of miasmata, he is well acquainted with the proper practice in the case.

There are several general laws with regard to miasmata, one or two negative ones are all important:—it is heavier than air; it is capable of being carried by the air within a certain distance; moisture, a certain degree of heat, and a mineral or vegetable agent or agents are necessary to its production; it cannot be produced beneath a certain temperature, and can be produced without the presence of vegetable matter; these, are all, with the exception probably of the last, in general credence with the profession; there are some medical gentlemen, however, who are even yet sceptical on the subject of miasmata. I have heard it denied that there is such an agent; the heat of the sun, or reflected heat; electricity, the presence of minute animalculæ in the air, or of fungi floating therein, have all had their advocates; it may be possible that the two last agents are the generating causes of this universal disseminator of disease; to mention the others to persons who have really seen many locations of miasmata, would be but to abuse their patience. We hold that moisture and heat operating on mineral, or some mineral or vegetable products, are necessary to the constitution of malaria; and in living at many places that were hot beds of intermittent fever, of congestive and bilious fevers *et id omne genus*, we have always found these present. The question then is as to the topography of the soil giving rise to these three agents. Let us take first, the description of a place that by every apparent circumstance ought to have been healthy.

Fort Macomb, Middle Florida, had the reputation in 1842 of being the most unhealthy in the territory. It was situated on the Sawannee, a rapid and deep river, navigable for a hundred miles above its mouth; the river opposite the fort had scarcely a single weed or decaying vegetable in it; it flowed at the rate of two and three-fourth miles an hour; its banks and bottom were formed of light granular disintegrating limestone, its waters were pleasant, had the taste of rain water, and like most of the rivers of Florida, were of a black colour, owing to infiltration at its source through pine and cypress roots; its banks did not shelve off gradually from the shore like most of the northern rivers, but became suddenly precipitous, and the river was often as deep five feet from the shore as it was in any part of the channel. I in vain endeavored to conjecture what could be the cause of the malaria so abundant about this Post; the quarters of the men were situated on a bluff thirty feet above the river, and about fifty feet from it. Among the soldiers the Post was called *The Hill*, and judging by the eye, I knew of no ground within many miles of it, which was of the same elevation. The river had been high, and its waters had been rising during nearly all the time we were at the Post; back of this Post, and around it, is a dry pine barren, extending for many miles, with not a marsh or anything approaching to the nature of one within four miles of the Fort, and then you meet with a small shallow lake, which, when its waters were nearly dry, might generate malaria, but it was at too great a distance, and the woods between it and the Post were too thick to lead one to suppose it was the source of disease. I had been around the Post for several miles in all directions; this pond, or lake, was the only one within that radius—even the hummacks on the other side of the river, of which there were two or three of a small size, were perfectly dry throughout their whole extent. The banks of the stream were high, nor did the river ever overflow them on either side. About the Fort, on the surface of the ground, the sand was almost a pure white, save here and there where some local causes had conspired to impart some fertility to it. The few dead branches of timber or decaying trees upon it were of the pitch-pine of the South, a tree that resists moisture and decay to a great degree, and which even in some cases seems to wear gradually away, more by the "soft handling of the elements" than by any cause of decomposition within. Speaking in general terms, there was no decaying vegetable matter about the place. A stranger would have said of it, "what a high, dry, and salubrious situation." Yet, even the *dogs* at this place had intermittent fever. I have repeatedly observed them; they would crawl out into the sun next the wall, become morose, chatter with their teeth, their whole bodies would quiver at intervals, and they would go through the phenomena of a regular stage of intermittent fever. I was ordered to this post in the month of December, when there was frost nearly every night. I had been but a few weeks in the Territory; I had come from the North, with a plus amount of health, and had never suffered from a day's sickness within my remembrance. Yet, ten days after I had set foot in this spot, (the thermometer during the day ranging from 45° to 80° and during the night from 29° to 45°), I was prostrated by a congestive fever, and for several days I vibrated between life and death, there were some sixty men there, some one-third of them in their beds in the hospital, and another third ailing in their quarters with *quotidian or tertian*

attacks of intermittent fever. Out of forty men who had been there the year before us, there were not men enough off the sick report to take care of the sick who were on it—it had previously been abandoned on account of the unhealthiness of the situation. For months I tried in vain to find out the cause of disease at this Fort. During December and January, and part of February, the quantity of rain that fell did not exceed an inch and a half, the ground on the surface was apparently dry and powdery, a great quantity of rain, however, had fallen in the previous summer.

I had observed that *fungi* flourished in great luxuriance about the Post—they were all, however, with one exception, of an unhealthy kind. I collected eighteen different specimens in a walk of two miles; one species grew very abundantly within some yards of the Hospital, and I was accustomed in my walk there, to make a detour to avoid this locality. This *fungus* had a most disgusting and nauseous odour, resembling more the smell of the fæces of a dysenteric patient than that of any other smell to which I can compare it; it was, I believe, a species of *Phyllus.* I have counted forty-two of these *fungi* within a space of fourteen yards square—the locality did not embrace more than an acre of ground, and was about a log building. The *fungus* lasts but thirty hours, but there was a fresh growth always on the spot, renewed apparently every morning. It is of a beautiful vermillion colour, save on the top, where a thick, black, molasses like secretion is deposited from the vegetable. So disagreeable was the smell, that where it was detected about a tent, or cabin, an active search was instituted, and floors raised, when the whole cause was often found to be but one *fungus.* I have never seen this fungus in any other place, North or South. As a general thing, it does not rain in Florida in the winter months. At Tampa Bay in December, January and February, of 1843, '44, the rain that fell only amounted to 5 7-10 inches, and that is a greater quantity than usually falls in those months, but fungi beyond all other vegetables require a great amount of moisture for their growth. Where then did they obtain it in this dry season? I was for a long time perplexed to find out, for I did not seek for moisture then in order to confirm any theory I had about malaria, but for the mere object of finding how those fungi supported their existence, and accident finally revealed it. The soldiers had occasion to sink a post into the ground near this locality, and I was surprised to find at the depth of two feet, a tough layer of putty-like clay, nearly as impervious to water as so much rock. I traced the stratum of this clay, and found it ran under the Fort, and for some distance beyond, at a depth varying from one to five feet; it had afforded the moisture indispensably requisite, I think, to the generation of that form of malaria producing periodic disease, but the vegetable matter, one other requisite according to the opinion of many, was undoubtedly wanting. This was not the only place, however, in Florida, where no decaying vegetable was present, and where periodic disease was rife. Fort Wacahoata, on the road from Pilatka, to Fanning, was in a high sandy pine barren, it was always esteemed one of the most unhealthy in Florida. I have never been more than a few hours at it, and consequently am unable to give any proper account, save this negative one of its situation.

The first Post I was ordered to in Florida, was Wacassassa, it was flanked on the eastern side by an extensive swamp or wet prairie, of some fifty acres in extent. This was rather a startling neighborhood for the latitude of Forida, and like all others who had examined the matter, I came to the conclusion that health was not one of the advantages to be possessed at Wacassassa. The very appearance of the swamp was loathsome; the water did not seem to be more than two feet deep in any part of it. It was surrounded by tall pines and oaks, preventing the breeze from blowing over it freely; nearly to the centre, the long heads of heavy water plants were to be seen, whilst along the margin for the distance of several yards, up to the edge of the water, the land was boggy and covered with a profuse growth of plants to be more pregnant with malaria; the road ran along its border, and in that dry season (it was in November,) was wet and muddy. The fort was built some three or four hundred yards from the centre of this marsh, but the outhouses extended down to the edges of it, and the trees were felled to such extent as to leave an open space with nothing to interrupt the currents of air, blowing directly over it, to the Fort. The buildings were some eight or ten feet above the water, but Wacassassa was one of the most healthy places in Florida, it was seldom that more than three or four were on the sick report from a command of fifty or eighty men, and these cases were generally casualies or diseases not incident to the country, periodical diseases were really wanting. Yet, this place, apparently so unhealthy afforded convincing proof of some of the most popular of the laws of malaria, there were ponds in every direction from it, as well as this chief one I have mentioned, but they were never *dry*, although very shallow in depth, and spread over a considerable surface. From some unknown cause unlike such collections of water generally in Florida, they retained in the centre even the dryest season of the year, enough of water to keep moist the whole margin; and, consequently the plants depending for their growth on a large share of moisture, retained their life through the year and were thus kept completely from decay. On the year after the commencement of the Florida war, this pond became completely dry, and the whole neighborhood suffered from severe intermittent disease; since then no region in Florida had been more healthy, for the pond had not been dry. A few miles from this Post is Payne's prairie, in the months in which this prairie is overflowed, the country around is healthy; when the waters subside, the reverse happens in a lamentable degree. I knew no exception in Florida, to the rule that a body of lowland submerged at one season of the year, and dry the remaining time, did not produce many serious cases of diseases on its borders, extending from a few yards to three fourths of a mile from the point of origin of the malaria, according to prevalent winds and the nature of intervening objects.

Fort Fanning on the Sawannee, was during all seasons, extremely unhealthy, below it, and opposite, across the river, were large cypress swamps, with many of the trees growing; but in part, the swamp was so covered with the dead trunks, that it was possible to cross it in nearly all directions on these prostrate and decaying trees. On entering New Orleans for the first time by the way of Lake Pontchartrain, I was struck with the resemblance of the extensive swamp between the city and the lake to these cypress swamps on the Suwannee. This swamp extends to the suburbs of the city, and is, I have no doubt, the cause of the disease so prevalent in it. I was compelled again to pass through New Orleans last October, when the yellow fever was so prevalent. I entered the city by the way of the Mississippi, and with true delight saw that the wind was blowing from the south. I walked through its almost deserted streets without fear, and was not astonished, on enquiry, to find that the wind had continued steadily in the opposite direction to that in which it was now blowing for several weeks before.

Tampa in Florida was one of the most healthy spots in the Territory; there were no marshes within two or three miles; the land shelved down to the water's edge by a gradual descent, so that the drainage of the ground was complete, the timber was cleared off for some distance about it, and to the most critical topographical eye it ought to have been, and was healthy. But a trip on the Hillsborough river, a sluggish stream that flowed by it with banks low and marshy some distance up, was an excursion not to be undertaken on slight grounds; unless you had previously been inured to malaric influence. One of the worst cases of congestive fever I have ever treated, happened to a person who made an excursion some two or three days before up this river; having entered the Territory by the way of Tampa and the sea, and remained sometime afterwards at Tampa before making this excursion, in perfect health. I had never seen this disease, or intermittent fever, occur in those who remained at the Post.

At Key West there was a marsh in the middle of the town, and the inhabitants suffer from yellow fever. The sick were removed to the Indian Key, a coral island, some five hundred yards in diameter, and several miles from the first named Key.

At Fort White, on the Suwannee, another eminently unhealthy post, there was a large, moist hummack, immediately across the river, some thirty yards in width. Here vegetable decomposion was going on to a very great extent.

At Charles' Ferry, on the same river, the only healthy spot I know upon it, the country was cleared for some distance about the house, and the land dry in the neighborhood.

At Cedar Key was the general hospital for the sick; this was a very small island, some four miles from the mainland; the beneficial effects of a removal to a pure atmosphere were probably never more apparent than in the cases sent from the pestiferous airs of the interior to this sea island; Sea-horse Key, in its vicinity, was also a depot for troops, and perfectly healthy.

In speaking of disease in Florida, and of these exhalations from swamps, from decaying vegetable matter, and from moist soil or sands were this matter was not present, I wish to be understood as referring principally to that form of malaria giving rise to *periodic disease* not only in the form of intermittent fever, bilious remittents, congestive, etc., but impressing its mark on every disease, that occurred in its locality. I have treated patients at Fort White and Fort Macomb, whose sole disease when asked what they were suffering from, I have termed the *malarial disease.* These men happened to be of that form of constitution which easily takes on malarial action, but in which every organ of the body was so nicely balanced that no one offered a salient point for attack; the nervous system was too well adjusted for an intermittent form of fever; the circulatory and nervous for a congestive seizure, and the chilopoietic for a bilious remittent; yet all three of these various actions would be disturbed—the appetite would fail, the slightest excess be followed by a deranged condition of the bowels: the strength so much weakened that a walk of a few hundred yards would be followed by prostration; the action of the heart would be so tumultuous and irregular, as frequently to induce the belief of severe lesion in that organ; any exposure to the sun would bring on a pain in the head; the body would become emaciated; the countenance sallow and cadaverous; if a whitlow or wound occurred in a patient of this kind, it would quickly take on regular periodic pains, yielding quickly to quinine. I have seen the heart in these patients so deranged that its flutterings could be easily perceived as you entered the hospital in the morning; and they have complained to me that, before "gun-firing" in the evening, they always had a chill. One of the worst cases of this disease I have ever seen, occurred in a young recruit, who had recently joined from the North. He was reduced to a shadow of his former bulk. I sent him to Cedar Key, and, some months afterward, being at the island, he came and spoke to me, as hearty a man as I saw on the island, and then employed as an attendant in the hospital. The cure was accomplished, as it only can be in these cases, by a removal from the cause of the disease. I am convinced there are few persons living in a highly malarious district, who do not suffer, for a time at least, from this malady. It is of many grades; and I have observed it, in officers, from depression of spirits, languor and lassitude, up to almost a complete cachectic disposition of body. This disease obtained, among the men, the name of "Suwannee Fever." But, although there were slight chills on almost any provoking cause, there was not generally any fever present, save when it assumed, as it occasionally did, a hectic form with night sweats. I immediately sent all these patients to the general hospital at Cedar Key, where they speedily recovered; but they they were generally useful in the Territory afterwards, and, in time, fell into the regular form of intermittent or other fevers.

The town of Pensacola was healthy; but the naval hospital, six miles from it, and the army barracks, or sheds rather, immediately in its vicinity, were very sickly—a marsh extended immediately in front of the hospital; and, by every fair mode of reasoning, it was generally considered the cause of the disease, so thoughtlessly, with regard to medical topography, was this hospital placed.

From Florida I went to Portland in Maine, a cold, bleak, unhealthy, but beautiful town. Its chief diseases, however, were those connected with the air passages from its immediate neighborhood to a stormy sea, and full exposure to eastern and northern winds, ladened with humidity; a stratum of a hard, slaty, gneiss-like character outcropping a great part of the surface, and nearly vertical, ran under the town and fort, and out to sea; it could be traced on land for more than a mile in thickness; the ground was high, and the latitude and situation such as to afford an insufficient heat for the generation of malaria.

From Portland I was ordered in the summer of 1846, to Holton, in the northern part of Maine, near the boundary of New Brunswick, and in sight of Mars' Hill. Here all the diseases depending on malaria were wanting, owing to the situation and latitude, and the peculiar nature of the country. The Fort near Holton was some 800 feet above the sea, and considerably elevated above the neighboring country, yet the rarity of the atmosphere, or some cause associated with elevation and pure air, imparted a peculiar complexion to disease in this latitude. I have seen the same effects on the western part of the country, in nearly the same latitude and elevation. These effects are somewhat assimilated to chills, or fraisons rather, with no fever following. They would occur often at regular periods during the day, and when unaccompanied by other disease had only the effect of rendering the patient uncomfortable. I have, usually, easily dispersed them with quinine.

From the extreme north-eastern I was ordered to the extreme north-western part of the Union, to a post a few miles west of Prairie du Chien, on the upper Mississippi, in the latitude of 43 degrees. This town, for the last few summers, has been eminently unhealthy, a large proportion of the cases proving fatal, running into a typhoid type, but still with high symptoms of congestion and periodicity. It is evident that the extensive marsh, stretching in front of the town, through the meadows between it and the Mississippi, is the cause of the disease. So every boy in the village will tell you, while the surrounding country is eminently free from disease. West of it, on a high prairie land, at Fort Atkinson, where I was stationed, the men enjoyed most excellent health. The few cases on the sick report being those of casualties, or of diseases that occur in any climate, or under any sun. This region suffers much more intense heat than the corresponding latitude on the East. At Holton, I had fires daily during the month of August, and frost was common at night; but I have not felt, even at Key West, more extreme heat than occurs in the summer months at Prairie du Chien and in the region of its neighborhood. North of this, at the falls of St. Anthony, in latitude 45 degrees, the Indians have suffered very much, within a few years, from the effects of malarious disease, and great numbers have died from intermittent fever. It seemed, when I was at this post, to be the general opinion that that fever had been known only among them within the last two or three years, at least it had never displayed itself in such violence as in these latter seasons. I am disposed to think some other cause was at work than the overflow of the Mississippi beyond its usual marks, for the periodic diseases have only, I am convinced, been common to the Indians of that neighborhoodwithin a very recent period.

Let us take a flight over to Mexico, and see the state of affairs there, not in a warlike, but a medico-topographical view. I landed at Brazos in May, and served for some weeks at Point Isabel, in sight of that island. Here a peculiarity was observed with regard to diseases of the intestines, and I doubt not many of those who have landed at the mouth of the Rio Grande or at Brazos have observed the same. I have seen it affect the men very seriously ; but I do not remember a case immediately fatal from it, though I am convinced it often ran into chronic dysentery. This disease was a dysentery of a very light grade, and one which the constitution generally surmounted in the course of a week or two, unless under peculiar circumstances. It was evidently produced from some atmospheric cause, for humidity was present elsewhere as here, the same food and water as bad. I was attacked with this dysentery in two days after landing at Brazos, (or rather at Point Isabel, having gone over to the post immediately;) in ten days, with very little care, it left me. Abstinence and blue pill and opium, were generally sufficient for its cure ; yet periodic disease, as far as my knowledge extended, was, I believe, unknown at that time in the neighbourhood. There are extensive flats in the vicinity of Point Isabel, constantly moist with the sea water, and large masses of sea-weed left exposed at low tide, often I am convinced in a state of decay from the beaming sun that came down upon them. From this post I was ordered to Monterey, and travelled to that city over the arid plains of Mexico, under the burning sun of June.

The want of rain, and consequently of moisture in the earth, will forever relieve the Mexicans from the curse of malarial disease, or of that form produced by exhalations from the earth's surface. I think it would be impossible for a country boasting of any vegetation whatever, to be drier than that between Camargo and Ceralvo. Even when you do occasionally meet with the course of a running stream, the water is often wanting, from the appropriation made of it by proprietors of land on its banks further up, who have drawn it all off for irrigating their lands. At Ceralvo you reach the spurs of the mountains, and water from their neighborhood becomes more abundant, though the land is still sterile and arid. At Monterey there is the bed, and occasionally part of the water of a large stream, and up the valley, at the distance of four or five miles, there is quite a volume of water, but it is generally all drawn off before it reaches the lower part of the city, for irrigating the land about it, or the gardens of the town. The ascending valley, between Monterey and Saltillo, is unfit for all purposes of cultivation, and is sterile to a lamentable degree. I remember but one spot, at the Rinconada Pass, where cultivation, to any extent, has been attempted, and there the land is well situated for irrigation. Saltillo is on a high table-like valley, between two ranges of mountains. The largest encampment in northern Mexico being in its neighborhood, on the skirts of the battle field of Buena Vista, the diseases from which the troops suffered most were from fevers assimilated to a typhoid type, dysenteries and diarrhœas. The exposure to the heavy dews of night, the diet, and excess in it, so often committed by soldiers, drunkenness, depression of mind from home-sickness, and from inactivity and hope deferred, peculiarities in the hygrometric condition of the atmosphere, and exposure to the rays of the sun, were also the fruitful causes, not only of the first-named diseases, but also of all others lying dormant in the system. Any disease which, under more propitious circumstances, might have remained inactive in the individual for life, would be sure to give some sign, under these circumstances. This is a most prolific cause of disease in all armies, and, chiefly so, when the recruit is not compelled to undergo a rigorous examination before enlisting. The diseases generally originating from these causes, are probably most often intermittent fevers, phthisis pulmonalis, or other diseases of the lungs, mesenteric disease, the development of the scorbutic diathesis, or some taint in the blood showing its existence by ulcers; disease of the joints, &c., rheumatism, nostalgia, &c. This principle, of all others, I think has been at the root of most of the cases of disease that have occurred in Mexico. I remember, a few days after I was stationed at Saltillo, of being ordered on a medical board, to ascertain, if possible, the cause of the disease at the camp at Buena Vista. There were some 500 sick; 280, if I remember aright, of that number being from the Carolina regiment. This sick squad—or at least upwards of 200 of them who were able to stand on their legs, were paraded in front of the regimental tents. I think, to any one who had seen much of malarious disease and the appearance of its victims, the cause of all this sickness would have been at once apparent. The board had no understanding or conversation of any kind, and made no investigations on this matter before this time, and the majority of the board had only been in Saltillo some few days, yet the very first question that was asked to the first man in the squad, by Dr. Byrne, showed the secret of the whole matter, and I was struck with the coincidence of his views and mine on the subject. "Well, my man," said he, "you have never had the fever and ague?" "Havn't I!" was the answer. "I've had it since I was *so* high." It had left its indelible marks on his whole outer man. We questioned every man of the regiment who was sick, and there was but one conclusion that could be arrived at—they had nearly, to a man, been sick from this disease, or some one of a like nature, before enlisting. They had generally been enlisted in a part of Carolina where miasmatic disease was very common; yet I do not remember of a single case of intermittent fever originating in Buena Vista or its neighbourhood, in which the disease had not occurred elsewhere in the individual. The astonishing tenacity of intermittent fever, and particularly its tendency to assume its empire again on account of the moisture in the atmosphere operating on the body, and from a multitude of other causes, are well known. I have known one case, in which it held its course, at intervals, for five years, the individual, within that time, having studiously refrained from approaching within any latitude or neighbourhood in which it was common; and, I am convinced, it often exists for much longer periods than this.

The encamping ground near Saltillo was on an inclined, rocky plain, about a mile from the foot of the mountain, with such an inclination that the water readily ran off. It rained here some three or four times in a week, from the immediate neighbourhood of the mountain, while in the town rain would fall but once or twice in the month. The mountainous ranges on either side, or the atmosphere about them, would form clouds, which were daily pouring forth their moisture, as you could see on their sides or summits, while the arid plains beneath would perish for the lack of water. Hence arose that peculiarity in the atmosphere which was so very prejudicial to health in Saltillo. The wind would come down from these mountains heavy with humidity, the sun would be obscured by the heavy, balloon-like clouds overhead, and perhaps a few drops of rain would fall, but generally the skies would become clear again in a few minutes' time, the sun would come forth with great power; the breeze would change, coming directly up or down the valley, with all its moisture absorbed by the arid plain it had travelled over. The nights were so cold and damp at the camp that two blankets were necessary, and overcoats in the morning, in the month of August, whilst the middle of the day was extremely warm. This was the history of nearly every day at Saltillo. Whether the state of the atmosphere, or some cause generated by it, or exposure, gave rise to the severe cases of typhoid fever and dysentery that were so common, I cannot say; but all diseases assuming a pure periodic type were wanting, save in those who had previously suffered therefrom.

That at the present hour we are close upon the discovery of what malaria really is, I am fully convinced. The investigations and theories of Liebig have pushed inquiries to the very door of this stronghold, so long closed to inquisitive, philanthropic, and scientific eyes. It remains for some future inquirer to lay open the subtle essence of this vigorous agent of death, which springs into existence in the noon-day. There is reserved for some coming investigator the glory of a discovery that will overtop in excellence all the results ever achieved by the labours of Jenner, of Watt, of Davy, Dalton, or of Franklin, to preserve the life, or advance the glory and comfort of mankind.

Very large portions of the earth's surface, and some of its richest soils, are rendered uninhabitable from the presence of this scourge. It selects its victims from every age and sex, from all kinds and conditions of men. It has swept off, by a thousand fold, more victims than have ever perished by war or famine; yet it is subject to the most simple physical laws, and was never, surely, intended to be always hidden from our view.

The results at which I have arrived from my own observation concerning the nature and *locale* of marsh miasmata may be summed up as follows:

It is generated at least as far north in the United States as the 45th degree of latitude, in the north-western States, and probably not so far north in the north-eastern.

Frost does not prevent its generation.

It is heavier than atmospheric air.

It is capable of being conveyed across a stream of at least thirty yards in width.

It is most generally found associated with heat, vegetable decomposition, and moisture.

Heat, and the continued percolation of moisture upwards through soil or sand, although vegetable decomposition is not present, in a hot sun, is capable of giving origin to malaria.

Vegetable decomposition, or the percolation of moisture through soil from sea water, does not seem to give rise to malaria.

I have seen* no exceptions to the rules, that meadow lands, low, level, and moist in the summer months; that the extensive decay of vegetable matter, with the constant upward progress of moisture, or the continued percolation of moisture through soil, or drying of extensive flats or marshes, did not, under proper heat, give rise to malaria.

A great degree of humidity *in the atmosphere*, on land, under a hot sun, not alone capable of originating malaria.

The effluvia giving origin to bilious, typhoid, and yellow fevers, to dysentery and diarrhœa, would seem to be essentially distinct from that which causes pure periodic disease, such as intermittent fever. Cases of the highest grade of dysentery and diarrhœa, however, are often associated with intermittent fever, when occurring epidemically.

The atmosphere being the vehicle by which malaria is disseminated, it is capable of being conveyed by it for probably the distance of three-fourths of a mile, there being no intervening objects to obstruct the wind; that it is not disseminated *against* the wind is evident.—*St. Louis Med. and Surg. Journal.*

* I am far from asserting there are no exceptions to these rules, but I have not happened, as far as I can recollect, to meet with any, in a tolerably extensive experience.

